# Comparative Effect of Incorporation of ZrO_2_, TiO_2_, and SiO_2_ Nanoparticles on the Strength and Surface Properties of PMMA Denture Base Material: An In Vitro Study

**DOI:** 10.1155/2022/5856545

**Published:** 2022-04-28

**Authors:** Emad Azmy, Mohamed Reda Zaki Al-kholy, Ahmad M. Al-Thobity, Mohammed M. Gad, Mohamed Ahmed Helal

**Affiliations:** ^1^Elmarg Students' Clinic, General Authority of Health Insurance, Western Elmarg Area, Cairo, Egypt; ^2^Department of Removable Prosthodontics, Faculty of Dental Medicine, Al-Azhar University, Cairo, Egypt; ^3^Department of Substitutive Dental Sciences, College of Dentistry, Imam Abdulrahman Bin Faisal University, P.O. Box 1982, Dammam 31441, Saudi Arabia

## Abstract

**Objective:**

This study aimed to investigate the effects of nanoparticles (zirconium dioxide (ZrO_2_), titanium dioxide (TiO_2_), and silicon dioxide (SiO_2_)) on the flexural strength, impact strength, hardness, and wear resistance of the acrylic resin denture base material.

**Materials and Methods:**

Acrylic resin specimens were fabricated in dimensions according to American Dental Association (ADA) specifications per test. Specimens were divided according to nanofiller into four groups; unmodified as control, ZrO_2_ (Z), TiO_2_, (T), and SiO_2_ (S) groups. Each one was subdivided into two subgroups according to nanoparticle concentrations; 3% and 7% (Z3, Z7, T3, T7, S3, and S7). A 3-point bending test, Charpy impact test, and Vickers hardness test were used for flexural strength, impact strength, and hardness measurements, respectively. Wear resistance was measured by the differences in surface roughness of tested specimens before and after the wear test. A scanning electron microscope was used to assess nanoparticle specifications and distributions and for fracture surfaces analysis. ANOVA, Bonferroni's post hoc test, and the Kruskal–Wallis test were applied for data analysis (*α* = 0.05).

**Results:**

Regarding the flexural and impact strength, there was a statistically remarkable increase for all tested groups compared with the control group, except for the T7 and S7 groups (*P* value <0.001, effect size = 0.893) and (*P* value <0.001, effect size = 0.759), respectively. There was a statistically significant improvement in the hardness of all tested groups compared with the control group (*P* value <0.001, effect size = 0.67) except T3 and S3. Regarding wear, a statistically significant enhancement was noticed in the wear resistance of all tested groups (*P* value <0.001, effect size = 0.685), except for the T7 and S7 groups.

**Conclusion:**

The flexural strength, impact strength, and wear resistance improved with both concentrations of ZrO_2_ and low TiO_2_ and SiO_2_ concentrations. The hardness increased with both concentrations of ZrO_2_ and high TiO_2_ and SiO_2_ concentrations.

## 1. Introduction

Polymethylmethacrylate (PMMA) is broadly utilized in the prosthetic rehabilitation of partially and totally edentulous individuals because of its acceptable aesthetics, ease of use, low cost, and stability in patient's mouth. However, insufficient mechanical properties and less fracture resistance are considered its major drawbacks as it was found that about 68% of complete dentures were liable to breakage during the first 3 years which may have occurred by either masticatory force or dropping a denture [1]. Many attempts were carried out to overcome PMMA's shortcomings and to enhance its biomechanical properties and clinical usage including modifications with metal plates or wires, rubber, metal oxides, and fibres. Recently, the evolutions in the field of nanotechnology as nanoparticles, nanofibers, and nanotubes were employed for PMMA reinforcement [2].

A significant concern has been raised on the adding of inorganic metal oxide nanoparticles to PMMA to enhance its characteristics, the previous studies reported that the properties of polymer nanocomposite depending on the type of incorporated nanofillers; their shapes and sizes as well as their concentration and interaction with polymer organic matrix [2]. The nanoparticles are characterized by their teeny size, huge specific surface area, as well as strong interfacial interaction with organic resin that leads to defining their unique mechanical, chemical, electrical, optical, and magnetic characteristics when compared to their bulk ones [3].

Amongst the commonly used nanoparticles are silicon dioxide (SiO_2_), titanium dioxide (TiO_2_), and zirconium dioxide (ZrO_2_). ZrO_2_ nanoparticles are ceramic materials having many advantages such as high strength, biocompatibility, and aesthetic acceptability compared with other metal oxide nanoparticles [3]. Many previous research studies have reported that incorporation of ZrO_2_ nanoparticles into PMMA denture base resin improved its mechanical properties [4, 5] dependent on ZrO_2_ concentrations [2]. Also, another study demonstrated that 5wt% of ZrO_2_ nanoparticles could enhance the mechanical and physical properties; fracture toughness and impact strength were significantly enhanced, as well as a remarkable decrease in water sorption and solubility [6].

TiO_2_ nanoparticles gained its importance due to its biocompatibility, low cost, corrosion resistance, and chemical stability with high strength [7]. It was reported that the addition of TiO_2_ nanoparticles to a polymer could affect its optical, chemical, and physical properties. In addition, its photocatalytic ability and antimicrobial effect encourage its addition to biomaterials [8, 9].

SiO_2_ nanoparticles were added to PMMA resulting in a noticeable positive effect on its mechanical and physical properties. Previous studies found that SiO_2_ nanoparticles added to PMMA denture base resins improved their mechanical properties [10–12]. Moreover, incorporating SiO_2_ nanoparticles in a low amount into PMMA resulted in improved strength, cracking resistance, and more durablity [13].

Different nanoparticles were investigated in the previous studies; however, there is a lack of comparative studies of these three nanoparticles within a single study. Therefore, this study aimed to evaluate the effects of 3wt.% and 7wt.% concentrations of ZrO_2_, TiO_2_, and SiO_2_ nanoparticles on the flexural strength (FS), impact strength (IS), hardness, and wear resistance of PMMA nanocomposite. The null hypothesis of this study was that the differences for the effects of the addition of different nanoparticles (ZrO_2_, TiO_2_, or SiO_2_) at different concentrations (3wt% and 7wt%) on FS, IS, hardness, and wear resistance of the heat polymerized PMMA would be insignificant.

## 2. Materials and Methods

The materials used in the current study and their manufacturing specifications are listed in Table 1. Heat polymerized acrylic resin was used to fabricate acrylic specimens in specific dimensions per test according to ISO 1567: 1999 for denture base polymers [14]. The rectangular stainless-steel plates with dimensions of 65 × 10 × 2.5 ± 0.03 mm, the rectangular stainless-steel plates with dimensions of 60 × 7 × 4 ± 0.03 mm, the rectangular stainless-steel plates with dimensions of 30 × 10 × 2.5 ± 0.03 mm, and the rectangular stainless-steel plates with dimensions of 20 × 20 × 3 ± 0.03 mm were prepared for FS, IS, hardness, and wear resistance, respectively.

**Figure 1:**
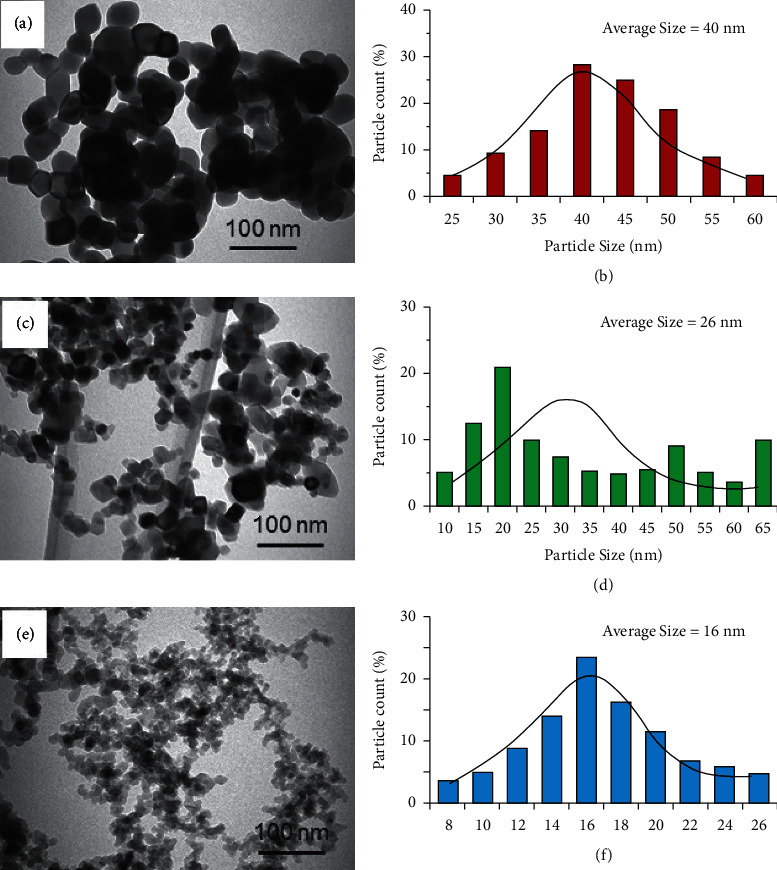
TEM and SEM representative images for nanoparticles utilized in the study, the average size of ZrO_2_, TiO_2_, and SiO_2_ particles is around 40 nm, 26 nm, and 16 nm, respectively.

The morphology, structure, and size of the nanoparticles were confirmed by transmission electron microscopy (TEM) (FEI, Morgagni, 268 at 80 kV) (Figure 1). The average size of the nanoparticles was found in the following order: 40 nm (ZrO_2_), 26 nm (TiO_2_), and 16 nm (SiO_2_). The dominant nanoparticles' shape was spherical, where as some hexagonal, cubic, and elongated particles were also observed.

ZrO_2_, TiO_2_, and SiO_2_ nanoparticles were treated separately by using a silane coupling agent as described in previous studies [2, 15]. A suitable amount of silanated nanoparticles was weighed by an electronic balance of 0.001gm accuracy (Denver Instrument, Göttingen, Germany) to be incorporated in 3wt.% and 7wt.% concentrations of acrylic resin powder. Each nanoparticle and acrylic resin powder were thoroughly mixed using mortar and pestle for initial blending followed by meticulously stirring for 30 min to ensure the homogeneity and distribution of the mix. According to nanoparticles, samples were divided into four groups: 3 modified groups (ZrO_2_, TiO_2_, and SiO_2_) and one control group (pure without filler). Furthermore, each group was subdivided according to nanoparticle concentrations, with subgroups modified with 3wt.% and 7wt.% nanoparticles of acrylic powder (*n* = 10). Based on previous studies, sample size calculation disclosed that a total of 280 specimens (70/test) were required to conduct the current study as shown in Table 2. All specimens were processed using a conventional water bath polymerization technique as described in the previous studies [5, 16].

For surface standardization, specimen polishing was completed using a cloth disc (TexMet C, PSA, 10in, Buehler GmbH) and a mechanical polisher (Metaserve 250 grinder polisher, Buehler) for 5 min at 100 rpm in a wet condition to avoid excessive heat which may lead to distortion of the specimens [17]. The accepted specimens were measured again with a digital caliper with an accuracy of 0.01 mm (Mitutoyo Corp, Tokyo, Japan). All samples were kept in distilled water (37 ± 1°C for 48 h) [18].

A flexural test was applied by using a three-point bending test with a universal testing machine (Model LRX Plus, Ametek Instruments, Fareham, England). Each specimen was horizontally mounted in a custom-made loading fixture with the aid of a jig on a computer-controlled material testing machine with a load cell of 5 KN. The load was set at zero then increased gradually until the specimen failed at a crosshead speed of 5 mm/min. At the point of fracture, the maximum force (N) was recorded and ﬂexural strength (FS) was calculated from the following formula [14, 19]:

(1) $$\begin{array}{c} FS\left(\sigma \right)=\frac{3Fl}{2wh^{2}}, \end{array}$$

where *F* is the maximum load (*N*) exerted on specimen, *l* represents the distance (mm) between two supports, *w* is the specimen width (mm), and *h* is the specimen thickness (mm).

The surface of fractured specimens was assessed by a scanning electron microscope (SEM) (SEM, TESCAN Vega3 LM model, Tescan Orsay Holding Kohoutovice, Czech Republic). The scanning was carried out after coating the specimens with gold (Quorum, Q150 R ES, UK) at an operating voltage of 20.0 kV. The SEM micrographs of specimens were recorded at different magnifications (x250, x500, x1000, and x2000) to assess the important surface features and set failure modes. The representative SEM micrographs of three reinforced PMMA specimens: ZrO_2_ (3% ZrO_2_ and 7% ZrO_2_), TiO_2_ (3% TiO_2_ and 7% TiO_2_), and SiO_2_ (3% SiO_2_ and 7% SiO_2_) were shown at a magnification of x1000.

For impact, specimens were prepared as previously described except that, by using a notch cutter (Notchvis; Ceast, Pianezza, Italy), a 3.5 mm notch was prepared at the midspan of each specimen. A Charpy-type impact tester (Beijing Jinshengxin Testing Machine Co., Ltd., Beijing, China) was used, in which the specimen was supported horizontally at each end, and the testing machine was adjusted at zero line. After that, the sample was stroked by a free swinging pendulum of 2 joules at the middle and on the side opposite to the notch. Impact speed was set to 2.9–3.5 m/s with 150° lifting angle [20, 21].

(2) $$\begin{array}{c} \text{IS}=\frac{E}{b\times d}, \end{array}$$

where (IS) is impact strength in (KJ/m^2^), *E* is the absorbed energy, *b* is the specimen width (mm), and *d* is the specimen thickness (mm).

A microhardness Vickers Tester (Laizhou Huayin Testing Instrument Co., Ltd., Model Hvs-50, China) with a diamond indenter and a 20X objective lens was used for hardness measurement. Five indentations with 200 g of load for 10 sec were applied on the specimen and then the average was calculated [22].

For the wear test, a two-body wear test was executed using a programmable logic-controlled machine; a 4-station multimodal ROBOTA chewing simulator integrated with thermocycling procedure operated on a servo-motor (ROBOTA chewing simulator, Model Ach-09075Dc-t, AdTech Co. Ltd., Germany). It includes four chambers to perform movements in horizontal and vertical directions simultaneously in thermodynamic conditions. Each chamber consists of an upper Jackob's chuck as a tooth antagonist holder and a lower plastic specimen holder in which the sample is embedded in a round Teflon housing by means of epoxy resin material. The test was repeated 10000 times, clinically simulating approximately one month of chewing function. Antagonist was attached to the upper member and prepared from natural teeth [23, 24]. In the current study, wear was measured by evaluating surface roughness before and after the wear procedure (Δ Ra) where the parameters of the wear test are mentioned in Table 3 [25, 26].

Quantitative analysis of two-body wear on the specimens and their antagonists was carried out before and after wear in a 3D-surface analyzer system [25, 27]. A digital microscope included a built-in camera (Scope Capture Digital Microscope, Guangdong, China) which was connected to a personal computer (Dell, Inspiron15, China) and was used to photograph specimens before wear simulation at a magnification of 120X [27]. The image was recorded at a resolution of 1280×1024 pixels/image. The digital image was cropped to 350 × 400 pixels using Microsoft Office Picture Manager (Microsoft Corporation, 14.0.2015, SP2) to standardize/specify the area of roughness measurement. The cropped image was analyzed by WS × M software (Ver5 Develop 4.1, Nanotec, Electronica, SL.) as all parameters related to the measurements were presented in pixels. Average heights in (*μ*m) were calculated using WSxM software as it is considered as a reliable index for the surface roughness. Consequently, the surface topography of each specimen was generated in a 3D image using a digital image analysis system (Image J 1.43 U, National Institute of Health, USA) where the unworn surface act as a reference. A 3D geometry of the worn surface was obtained, then the 3D images were collected for each specimen and the mean surface roughness (*µ*m) was calculated by averaging three readings on each specimen (at the central and both sides) [25, 27].

After wear simulation, the testing device was stopped; the sample's surface was cleaned with a brush to remove any surface particles or debris. Each specimen was photographed again as before to record Ra2. The change in surface roughness measurements before and after the wear simulation that occurs in each sample was determined according to the equation:

(3) $$\begin{array}{c} \Delta \text{Ra}=\text{Ra}2-\text{Ra}1. \end{array}$$

Statistical analysis was conducted by IBM SPSS Statistics for Windows (Version 23.0. Armonk, NY: IBM Corp.). The data were assessed for normality by evaluating its distribution and performing normality tests (the Shapiro–Wilk and Kolmogorov–Smirnov tests). All the data showed a parametric (normal) distribution. The data were presented as the mean and standard deviation values. A 1-way-ANOVA test was applied for comparison between all groups. Bonferroni's post hoc test was carried out for pair-wise comparison when ANOVA test is significant. The significance level was set at *P* < 0.05.

## 3. Results

The mean, standard deviations (SD), and significant difference between groups for all tested properties are summarized in Table 4. A statistically substantial difference was noticed between the FS of the different groups (*P* value <0.001, *Eta squared* = 0.893). All reinforced groups had significantly higher flexural values when compared with the V0 (control) group, except the T7 and S7 groups. An insignificant difference was found between Z3, Z7, and T3; all showed statistically significant highest mean FS values, while S3 showed a significantly lower mean value.

**Figure 2:**
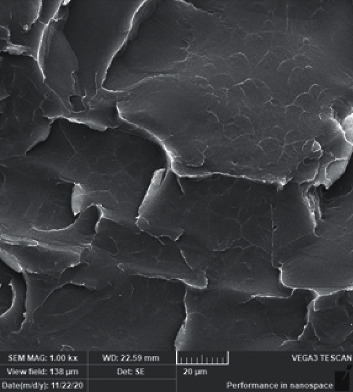
Representative SEM image for the control group.

**Figure 3:**
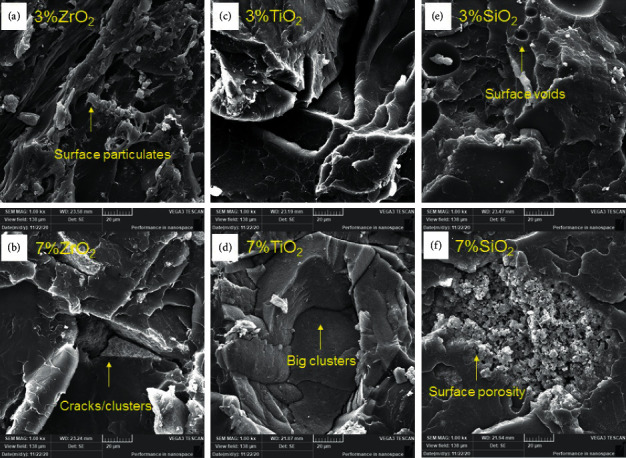
(a–f) Representative SEM images for nanoparticles-modified specimens: (a) Z3, (b) Z7, (c) T3, (d) T7, (e) S3, and (f) S7.

Figure 2 shows SEM for the control group and display a smooth background with small and faint lamellae which represent brittle fracture type. A dramatic change in the surface topography of the fractured surface of nanoparticles reinforced specimens, as shown in Figure 3, with ZrO_2_, more irregular lamella with well dispersion of nanoparticles within resin matrix (Figure 3(a)), while less lamella with faint steps and clusters formation was displayed with Z7 (Figure 3(b)). Also, T3 showed more lamellae and wide depressions with homogenous distribution of nanoparticles (Figure 3(c)), while the surface topography changed to faint lamella forming a wide groove and small clusters at the groove borders (Figure 3(d)). Voids and smooth surface with slight disappearance of irregularity were exhibited with 3% SiO_2_ (Figure 3(e)) and this future increased with S7 in addition to large clusters formations (Figure 3(f)).

As shown in Table 4, the IS of different groups appeared to be significantly different (*P* value <0.001, *Eta squared* = 0.759). The statistically significant highest mean is shown in the Z7 group. Insignificant difference was found between Z3, T3, and S3; all showed lower mean IS values. An insignificant difference was reported between V0 (control), T7, and S7; all showed the lowest mean IS values.

As shown in Table 4, there was a substantial difference between the hardness of different groups (*P* value <0.001, *Eta squared* = 0.67), where Z7 showed statistically significant highest mean hardness. An insignificant difference was presented between Z3, T7, and S3; all showed the statistically significant lower mean hardness values. T3 showed a statistically significant lower mean value with a nonstatistically significant difference from all other groups except Z7. Insignificant difference was noticed between V0 (control) and S3; both showed statistically significant lowest mean hardness values, with a nonsignificant difference from T3 but with a statistically significant lower mean value compared to all other groups.

As shown in Table 4, a remarkable difference was found between ΔRa of the different tested groups (*P* value < 0.001, *Eta squared* = 0.685). A substantial difference was noticed between V0 (control), Z3, Z7, T3, and S3, where Z3, T3, and S3 showed statistically significant lowest mean ΔRa values. There was no statistically significant difference between T7 and S7; both showed the highest significant mean ΔRa values. An insignificant difference was found between Z7, T7, and S7.

## 4. Discussion

In the present study, three different nanoparticles (ZrO_2_, TiO_2_, and SiO_2_) were selected due to their best unique physical, mechanical, and optical properties [7, 28–33]. Previously, the tested concentrations of the nanoparticles ranged from 0.5% to 10%. This huge variation presented an argument about the effect of the nanoparticles on the mechanical properties of denture base resin. Generally, low concentrations showed a favorable effect while high concentrations had a negative effect [7, 31–33]. In addition, it was reported that nanoparticle concentrations above 7% could cause a remarkable change in the color of nanocomposite [7, 34]. Thus, 3% and 7% were chosen to relatively represent both low and high concentrations [16, 28, 31]. Based on the results of the current study, the addition of ZrO_2_, TiO_2_, and SiO_2_ nanoparticles affected all tested properties; therefore, the null hypothesis was rejected.

The findings of the current study showed that the FS increased with ZrO_2_ nanoparticle addition and that the increase was concentration dependent which may be referred to the uniform distribution of too small sized ZrO_2_ nanoparticles used in this study, which enabled them to fill spaces between linear chains of polymer matrix, resulting in restricting the segmental motion of macromolecular chains which increased the fracture resistance with enhanced flexural strength [5, 28]. Also, the increase in FS may be because of the transformation toughening of ZrO_2_; when sufficient stresses were developed and the microcrack began to propagate, the ZrO_2_ nanoparticles transformed from tetragonal to monoclinic crystalline, depleting the energy of the microcrack and arresting its propagation. Furthermore, expansion of ZrO_2_ crystals occurred, placing the crack under compression state leading to stopping its propagation [35]. These findings were in line with several studies [36–38]. On the contrary, Ergun et al. investigated various ratios (5, 10, and 20wt.%) of ZrO_2_ to heat-cured PMMA and noticed that FS was reduced with the increase of ZrO_2_ concentration [29].

The addition of 3wt.% of TiO_2_ nanoparticles increased the FS which may be referred to the well dispersion of TiO_2_ nanoparticles in PMMA matrix at low concentrations [39]. When TiO_2_ passes into the matrix, it minimizes the mobility of the polymer chain because of strong interfacial interactions between the PMMA matrix and the nanofiller [40, 41]. While 7wt.% TiO_2_ decreased the FS in comparison to 3wt.%, this reduction could be explained based on the fact of TiO_2_ nanoparticles agglomeration and clusters formations at high concentrations, these clusters may act as stress concentration areas leading to weaken the FS [2, 7].

The addition of SiO_2_ nanoparticles led to an increase in the FS at low concentrations (3wt.%). This was in agreement with previous articles which reported that the addition of low SiO_2_ led to an increase in the FS of modified PMMA and repaired denture bases, providing better mechanical properties compared with its high content [42, 43]. This improvement in the FS may be due to the homogenous allocation of nanofillers and their capability to penetrate spaces in the interpolymeric chain and control their movement [42, 44]. In addition, the silane treatment allowed SiO_2_ to form a strong bond with the polymer matrix and enhanced the interfacial shear strength between the resin matrix and inorganic nanoparticles owing to cross-linking or supra molecular bonding which prevented crack propagation, thus enhancing mechanical interlocking [27, 45]. While SiO_2_ concentration was increased from 3wt.% to 7wt.%, the FS was decreased and showed the lowest value between nanoparticles-modified groups which may be justified by SiO_2_ nanoparticle aggregation and cluster formation leading to weak bonding and stress concentration [13, 42]. This outcome was in agreement with previous studies conducted by da Silva et al. [46] and Sodogar et al. [10], who found similar results to that of this present study. Also, Balos et al. [13] reported that low SiO_2_ nanoparticle concentration provided superior mechanical properties.

The findings of the present study reported variable effects between different nanoparticles on the FS of nanoparticles-modified PMMA, where 7% ZrO_2_, 3% ZrO_2_, 3% TiO_2_, and 3% SiO_2_ showed reasonable values above ADA recommendation values for FS (65 MPa), while other reinforced and control groups reported low values than recommended [18].

In the current study, reinforcement with ZrO_2_ caused significantly higher IS and increased as the concentration increased. This increase could be linked to the smallest particle dimension and uniform distribution of ZrO_2_, which could cause an increase in crack elongation during the process of fracture which may lead to an increase of energy absorption before fracture [37]. In agreement with the findings of the current study, Ebrahim et al. found that ZrO_2_ nanofillers incorporated into PMMA have a positive impact on IS and the best mechanical properties are obtained by adding a 7%wt ZrO_2_ concentration [31].

This finding was in disagreement with Begum et al. [30] and Gad et al. [16], who reported that IS was significantly reduced as the concentration of ZrO_2_ nanoparticles increased. Furthermore, in disagreement with Zidan et al., who reported a decrease in IS with all tested concentrations in comparison to the control group [28]. The difference in results may be attributed to denture base resin and material type where Gad et al. [16] used cold-cured repair resin material while Zidan et al. [28] used high impact acrylic resin.

Regarding the nano-TiO_2_ effect on IS, the addition of TiO_2_ nanoparticles to heat-cured PMMA resulted in a positive effect compared to unmodified PMMA [47]. In coincidence with the present findings, Aziz [48] reported that the addition of 3% TiO_2_ showed an improvement in the impact strength of acrylic resin denture base. Also, several studies have confirmed these findings with different concentrations of nano-TiO_2_, including 1wt.% [41, 49], 2wt.%, and 3wt.% [50, 51]. The same result was reported after the addition of treated TiO_2_ nanoparticles [48]. The improvement in IS was explained by the presence of an adequate bond between the PMMA resin matrix and nano-TiO_2_ which leads to restriction of segmental motion. Furthermore, the large surface area of teeny small particles helps in energy dissipation [48]. Other articles reported that the nanoparticles in PMMA resin bear most of the applied load while the resin matrix aids in structural integrity and distribution of the load, which ultimately reduces crack propagation [49].

Although SiO_2_ nanoparticles have been investigated in many studies, there is a lack of information about their effect on the IS of modified PMMA nanocomposite, making the comparison with previous studies difficult. The IS increased with 3wt.% SiO_2_ nanoparticles and this increase may be attributed to interfacial shear strength between resin matrix and nanoparticles owing to cross-linking or supramolecular bonding which arrests crack propagation [42]. While the IS decreased with 7wt.% SiO_2_ nanoparticles due to the large loosely clusters of agglomerated SiO_2_ nanoparticles, this was in accordance with previous studies that reported that low concentrations of SiO_2_ nanoparticles improved the IS while it decreased with the high concentrations [44, 52].

In the current study, an obvious improvement in hardness was achieved with all nanoparticles in comparison to control, although some groups was not significant. Moreover, 7wt.% showed higher hardness values compared with 3wt.%. This increase may be referred to the presence of nanoparticles on the specimen surface and good bonding between nanofiller and resin matrix, which requires more energy to break this bond [45]. The outcomes of the present study were similar to the results of previous studies that found that incorporation of different nanoparticles into PMMA resin significantly improved surface hardness [13, 16, 28, 39, 43, 47–49, 53]. Contrary to the findings of the present study, Cevik et al. [54] reported that SiO_2_ nanoparticles insignificantly increase the hardness of the PMMA denture base. Also, the findings of this study were in disagreement with those of da Silva et al. [46], who found that incorporation of surface-treated SiO_2_ at concentrations of 0.1–5wt.% resulted in reduced hardness of modified PMMA nanocomposites.

Based on the results of the present study, the wear resistance of 3% ZrO_2_, 7% ZrO_2,_ 3% TiO_2_, and 3% SiO_2_ modified PMMA groups was significantly higher than the control and other reinforced groups. This may be attributed to the exciting strong bond as a result of chemical interaction between nanoparticles and resin matrix which is considered the main cause of wear resistance of reinforced groups. Additionally, this also reduces the incidence of nanoparticles exfoliation during abrasion [55]. Duraid et al. stated that the addition of ZrO_2_ nanoparticles (3 and 5wt.%) improved the wear resistance of PMMA denture base material which may be explained mainly by the physical properties of nano-ZrO_2_, such as hardness and density, which might allow them to maintain their surface integrity and retain a highly smooth surface [56].

Zhang et al. found that PMMA nanocomposites had the highest wear resistance when TiO_2_ content was about 3wt.%. Furthermore, the surface of a nanocomposite was reported to be smoother [57]. Muhammad et al. observed that SiO_2_ and TiO_2_ fillers improved the wear resistance of artificial teeth [58]. On the other hand, Helal et al. studied the effect of SiO_2_ nanoparticles (0.1, 0.3, and 0.5wt.%) of denture teeth and reported a reduction in the wear resistance of PMMA denture teeth [59]. Also, in contradiction with the present results, Monadle et al. concluded that adding untreated nano-ZrO_2_ did not increase the abrasive wear resistance of PMMA [60]. This controversy between the outcomes of the present study and other studies may be attributed to nanofiller type, size, shape, concentrations, or mode of addition, as well as differences in the methodology, such as wear type, configuration of specimens, and simulator type used.

From the clinical point of view, modification of PMMA with inorganic nanoparticles, such as ZrO_2_, TiO_2_, or SiO_2_ nanoparticles, to improve its mechanical properties such as fracture and abrasion resistance and hardness has benefitted in some dental applications such as removable dentures and occlusal splint appliances. However, there are some limitations to this study related to inaccurate prediction of clinical performance of tested materials, as under clinical conditions, numerous additional factors such as presence of saliva, dietary habits, neuromuscular force, parafunctional habits, and different cleansing protocols can influence the results, so the presented findings are only a promising starting point for further investigations. Furthermore, the use of a simple rectangular-shaped specimen did not reflect the shape of an actual denture. In addition, only one type of denture base material was tested.

## 5. Conclusions

Incorporation of 3% ZrO_2_, 7% ZrO_2_, 3% TiO_2_, and 3% SiO_2_ nanoparticles significantly increases the flexural, impact strength, and wear resistance of PMMA acrylic resin. Also, incorporation of 3% ZrO_2_, 7% ZrO_2_, 7% TiO_2_, and 7% SiO_2_ nanoparticles significantly increases the hardness of PMMA acrylic resin. A 7% concentration of ZrO_2_ and TiO_2_ may be beneficial in preventing denture fractures resulting from clinical use, while SiO_2_ is recommended in low concentrations (3%).

## Figures and Tables

**Table 1:** The study's materials.

Trade name	Manufacturer	Specifications
Vertex	Vertex Dental, Netherlands	Powder: polymethyl methacrylate, 500gm
		Liquid: phthalyl butyl glycolate, ethanol, 250 ml
ZrO_2_ nanoparticles	NanoGATE, Ciro, Egypt	Spherical, white, and tetragonal particles (12 ± 3 nm; purity >99%)
TiO_2_ nanoparticles	NanoGATE, Ciro, Egypt	Spherical, white, and anatase particles (15 ± 3 nm; purity >99%)
SiO_2_ nanoparticles	NanoGATE, Ciro, Egypt	Spherical, white, and amorphous particles (21 ± 3 nm; purity >99%)
Silane coupling agent	Sigma-Aldrich Chemie GmbH	Purity 98%, ethanol 99.7%, lot no. 440159
	Riedstrasse2, Germany	

**Table 2:** Coding of different subgroups.

	Group	Code	Description
No. 1	Control	V0	Unreinforced heat cured acrylic resin

No. 2	ZrO_2_	Z3	Heat cured acrylic resin reinforced with 3wt.% of ZrO_2_ NPs
		Z7	Heat cured acrylic resin reinforced with 7wt.% of ZrO_2_ NPs

No. 3	TiO_2_	T3	Heat cured acrylic resin reinforced with 3wt.% of TiO_2_ NPs
		T7	Heat cured acrylic resin reinforced with 7wt.% of TiO_2_ NPs

No. 4	SiO_2_	S3	Heat cured acrylic resin reinforced with 3wt.% of SiO_2_ NPs
		S7	Heat cured acrylic resin reinforced with 7wt.% of SiO_2_ NPs

**Table 3:** Wear test's parameters.

Cold/hot bath temperature (5°C/55°C)	Dwell time (60 sec)
Vertical movement: 1 mm	Horizontal movement: 3 mm
Rising speed: 90 mm/s	Forward speed: 90 mm/s
Descending speed: 40 mm/s	Backward speed: 40 mm/s
Cycle frequency: 1.6 Hz	Weight/sample: 700 mg
Torque: 2.4 N.m	

**Table 4:** 1-way ANOVA and pairwise comparisons tests between different acrylic resin subgroups for all tested properties.

	FS (MPa)	IS (KJ/m^2^)	Hardness (VHN)	ΔRa (*µ*m)
Group	Mean ± SD			
V0 (control)	59.4 ± 5.5^C^	1.78 ± 0.21^C^	37.9 ± 1.4^C^	0.0025 ± 0.0002^C^
Z3	82.4 ± 5.8^A^	2.60 ± 0.39^B^	41.1 ± 1.1^B^	0.0016 ± 0.0003^A^
Z7	87.3 ± 2.2^A^	3.3 ± 0.31^A^	44.4 ± 1.3^A^	0.0021 ± 0.0002^B^
T3	83.4 ± 3.2^A^	2.26 ± 0.32^B^	39.9 ± 1.1^BC^	0.0017 ± 0.0002^A^
T7	62.2 ± 2.5^C^	1.97 ± 0.22^C^	41.3 ± 1.8^B^	0.0023 ± 0.00002^BC^
S3	70.3 ± 2.6^B^	2.45 ± 0.33^B^	38.8 ± 1.4^C^	0.0017 ± 0.0001^A^
S7	60.2 ± 5.0^C^	1.85 ± 0.21^C^	41.1 ± 1.6^B^	0.0023 ± 0.0003^BC^
*P*value	*P* < 0.01^*∗*^	*P* < 0.001^*∗*^	*P* < 0.001^*∗*^	*P* < 0.001^*∗*^
Effect size (*eta squared*)	0.893	0.759	0.67	0.685

^*∗*^Significant at *P* < 0.05, different superscripts vertically indicate statistically significant difference between groups.

## Data Availability

The data are available from the corresponding author upon reasonable request.
